# Response to article by Matthew Wasserman et al. (2018): “Modeling the sustained use of the 13-valent pneumococcal conjugate vaccine compared to switching to the 10-valent vaccine in Mexico”

**DOI:** 10.1080/21645515.2018.1554974

**Published:** 2018-12-20

**Authors:** Jorge A. Gómez, Adriana Guzman Holst, Javier Nieto

**Affiliations:** a Health Outcomes Department, GSK, Victoria, Buenos Aires, Argentina; b Health Outcomes Department, GSK, Panama City, Panama; c Medical Affairs Department, GSK, Panama City, Panama

**Keywords:** Cost effectiveness, Pneumococcal vaccines, Immunization Program, Mexico

## Abstract

In a recent article, Wasserman et al. estimated and forecasted the health and economic impact of switching from the 13-valent (PCV-13) to the 10-valent (PHiD-CV) pneumococcal conjugate vaccine in Mexico’s national immunization program. In this response letter, we highlight various methodological inconsistencies and model input considerations that potentially bias the results and further recommendations made by the authors.

Dear Editor,

In a recent article, Wasserman et al. estimated and forecasted the health and economic impact of switching from the 13-valent (PCV-13) to the 10-valent (PHiD-CV) pneumococcal conjugate vaccine in Mexico’s national immunization program.^1^ In this response letter, we highlight various methodological inconsistencies and model input considerations that potentially bias the results and further recommendations made by the authors.

The model used by Wasserman et al., previously applied in the context of Canada,^2^ forecasted future pneumococcal disease trends based on historical serotype behaviors for each PCV-13 serotype and non-vaccine serotypes. Throughout the manuscript, there appears to be no recognition regarding the time-period PHiD-CV was used in the Mexican Social Security Institute (IMSS), as it only mentions its registration and availability through the private market. They retrospectively analyzed the cost-effectiveness of pneumococcal conjugated vaccine (PCV) introduction in Mexico and described the existence of PCV-7 and PCV-13 between 2006 and 2014. Their one-sided and highly limited focus narrowly addresses the sequence from PCV-7 to PCV-13 and they improperly assume that all the health benefits observed in this time period are associated with those vaccines. Noticeably absent is any attribution or acknowledgement that PHiD-CV was used in the IMSS between January 2010 and December 2011 with approximately 2.2 million doses distributed.

Wasserman et al. used a simple liner and logistic regression model to simulate and forecast the complex behavior of pneumococcal serotypes prior to and post vaccine introduction in Mexico. To effectuate a reliable and valid health economic analysis, there are rubrics regarding the biological process that should be enclosed in the models to reflect how infections, demographic mortality, protection against infection, costs and use of healthcare resources occur over time.^3^ In the analytical framework used by Wasserman et al., comparisons between the model’s estimations and past data observations are not clearly presented due to limited explanation of how well trend regressions could predict historical data (see figure 2 in Wasserman et al.).^1^

It is important to emphasize that investigators affiliated to recognized public health entities such as the Pan-American Health Organization (PAHO), the International Vaccine Access Center (IVAC), and the World Health Organization (WHO) have each conducted independent systematics reviews which concluded that there was no superiority of one vaccine over the other.^4–6^ Therefore, the conclusion of the analysis of Wasserman et al. is inconsistent with the evidence already generated. Additionally, PAHO and WHO support the interchangeability of these vaccines in certain cases of programmatic and resources issues.^6^ While the results of Wasserman et al. suggest that continued use of PCV-13 instead of switching to PHiD-CV would likely save 34 Billion MXN over the next 10 years, the analysis appears significantly biased considering an invasive pneumococcal disease (IPD) serotype-specific approach. The evidence of cross protection against serotype 19A provided by PHiD-CV and the heterogeneity of effectiveness/impact results on serotype 3 observed with PCV-13 appear not to have been considered,^7–9^ misinterpreting the outcomes and conclusions. It is important to note the serotype content of PCVs may not automatically translate into disease protection against these serotypes and the absence of a certain serotype will not automatically translate into the absence of an effect due to cross protection.^7–9^

The serotype-specific regression analysis is designed to forecast future disease trends based on historical serotype behaviors. Due to the uncertainty surrounding serotype replacement and to avoid under-estimation or over-estimation of vaccine impact, tests on several scenarios were conducted by varying trend lines based on historical surveillance data from the UK and USA to reflect PCV-13 infant vaccination. Nevertheless, it is not clear how the prevalence of serotype 19A was considered under the Mexican scenario after PCV-13 introduction. According to SIREVA Laboratory Surveillance Network and National Institute of Public Health (INSP) data,^10,11^ serotype 19A prevalence tends to reduce after PCV-13 use, yet it still circulates following a secular pattern and it remains the most important invasive serotype in the Mexican setting.

Finally, there are certain weaknesses with the data inputs and assumptions made for clinical outcomes. First, the model presented by Wasserman et al. relies upon the historic demonstration of serotype-specific vaccine effect against IPD. Surprisingly, the authors fail to consider the evidence of PCVs efficacy against pneumonia and acute otitis media (AOM). Contrarily, the authors improperly estimate the prospective change in pneumonia and AOM cases based on the forecasted change for IPD cases.

We recognize the efforts of the authors to develop tools that evaluate the epidemiological scenarios and management costs for pneumococcal diseases, but these models should incorporate all available evidence showing the impact of PCV on overall pneumococcal disease instead of focusing solely on an arbitrary selection of evidence and assumptions. Vaccine impact estimations and cost-effectiveness data are crucial to inform policy-makers on PCV use. Considering all these general concerns, the Wasserman et al. study should be interpreted with caution.
